# Editorial: The Role of Bioactive Lipids in Homeostasis and Pathology

**DOI:** 10.3389/fphys.2021.773632

**Published:** 2021-11-22

**Authors:** Chunjiong Wang, Jun Yang, Xu Zhang

**Affiliations:** ^1^Department of Physiology and Pathophysiology, The Province and Ministry Co-sponsored Collaborative Innovation Center for Medical Epigenetics, Tianjin Medical University, Tianjin, China; ^2^Department of Entomology and Nematology, University of California, Davis, Davis, CA, United States

**Keywords:** bioactive lipid, eicosanoid, oxylipin, sphingolipid, lysophospholipid, metabolic diseases, cardiovascular diseases, respiratory diseases

Bioactive lipids, including fatty acids and their metabolic products, acylglycerol derivatives, endocannabinoids, lysophospholipids, sphingolipids, and cholesterol metabolites, etc., play active roles in regulating cellular functions. They are not only products of lipid metabolism, but also important signals in tissue homeostasis and pathology. The functions of a large number of bioactive lipids remain unclear. Moreover, even the same lipid mediator reveals various functions by activating various receptors. Thus, it is important to clarify the receptors and signal transduction capabilities of these lipids. The aim of the current Research Topic is to provide a thorough overview of the function, signal transduction, and regulatory mechanisms of bioactive lipids. This Research Topic will provide insight into their effects on homeostasis and pathology. The current Research Topic includes six reviews and five original research articles. These studies focus on the effects of bioactive lipids in the homeostasis and pathology of the cardiovascular system, respiratory system, liver, and adipose tissue dysfunction as well as others (Figure 1).

**Figure 1:**
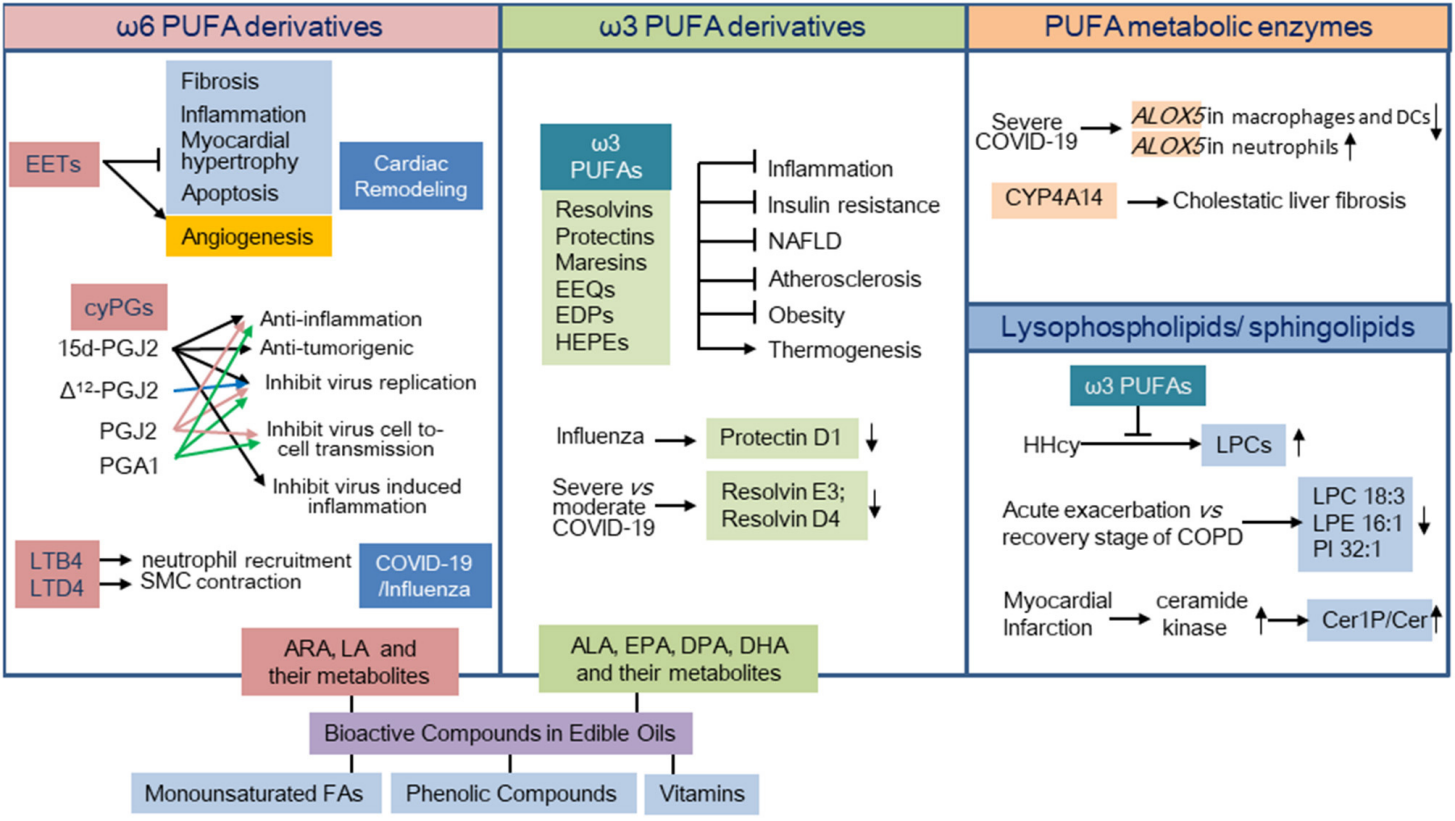
Overview of the contributions included under the Research Topic “Role of Bioactive Lipids in Homeostasis and pathology.” PUFA, polyunsaturated fatty acid; CyPGs, cyclopentenone prostaglandins; ARA, arachidonic acid; LA, linoleic acid; ALA, alpha-linolenic acid; EPA, eicosapentaenoic acid; DHA, docosahexaenoic acid; DPA, docosapentaenoic acid; FA, fatty acid; HHcy, hyperhomocysteinemia; LPC, lysophosphatidylcholin; LPE, lysophosphatidylethanolamine; PI, phosphatidylinositol; Cer1P, ceramide-1-phosphate; Cer, ceramide; SMC, smooth muscle cell; DC, dendritic cell. See text for a brief description and the reference of each contributing article.

Arachidonic acid-derived eicosanoids, including prostaglandins (PG), thromboxanes, leukotrienes, lipoxins, hydroxyeicosatetraenoic acids, and epoxyeicosatrienoic acids (EETs), among others, have been the focus of serious interest due to their vital roles in many physiological and pathophysiological processes. The roles of EETs and metabolites of arachidonic acid by cytochrome P450s were reviewed by Lai and Chen. This review suggested that increasing the levels of EETs is a potential therapeutic strategy for cardiovascular disease (Lai and Chen). Cyclopentenone prostaglandins (cyPGs) are a cluster of PGs with a cyclopentenone ring structure. CyPGs (PGA1, PGA2, and PGJ2 and its' metabolites) biosynthesis, mechanism of action, functions, and their effects on virus infection and cancer development were discussed in a review by Lee et al. In addition to arachidonic acid-derived eicosanoids, bioactive metabolites of ω-3 polyunsaturated fatty acids (PUFAs) have also drawn interest in recent years. Duan et al. summarized the effects of ω-3 PUFA-derived oxylipins on metabolic disorders, including diabetes, non-alcoholic fatty liver disease, adipose tissue dysfunction, and atherosclerosis. This review highlighted the importance of these derivatives when exploring the therapeutic effects of eicosapentaenoic acid (EPA) and docosahexaenoic acid (DHA) (Duan et al.). Moreover, an original research article reported that ω-3 PUFA treatment improved HHcy-induced insulin resistance and inflammasome activation in adipose tissue. HHcy increased lysophosphatidylcholine (LPC) 16:0 and LPC 18:0 levels in adipose tissue, which were suppressed by ω-3 PUFA treatment. This study linked ω-3 PUFAs to lysophospholipid production (Li et al.). As the major components of edible oil, the roles of ω-6 PUFAs (arachidonic acid and linoleic acid), ω-3 PUFAs (EPA, DPA, DHA, and alpha-linolenic acid), their metabolites, and the role of monounsaturated fatty acids in oxidative stress and inflammation were reviewed by Mazzocchi et al. In addition, this review article discussed the clinical studies conducted with various seed oils and marine animal-derived oils. This review highlighted the fact that high heterogeneity in oil composition plays a significant role in health outcomes (Mazzocchi et al.). In addition, as reported by Liu et al., the metabolite profiles of arachidonic acid and DHA are altered in high iodide-intake-induced hypothyroid offspring rats. Iodide intake adjustment plus 1,25(OH)2D3 supplementation ameliorated hypothyroid and metabolic disturbances along with increased serum EET and hydroxyeicosatetraenoic acid (HETE) levels (Liu et al.).

The effects of bioactive lipids in the respiratory system were also emphasized in this Research Topic. A clinical study reported changes in serum glycerophospholipids in the acute exacerbation of chronic obstructive pulmonary disease (COPD) and various subtypes of COPD (Gai et al.). Sahanic et al. thoroughly reviewed bioactive lipid mediators in COVID-19 and influenza. They further promoted the current knowledge regarding the mediator lipidome in severely affected COVID-19 patients. They accomplished this by investigating a publicly available RNA-seq database of bronchoalveolar lavage cells (Sahanic et al.).

The effects of sphingolipids on the cardiovascular system were reported in a research article from Hua et al. The authors demonstrated that the myocardial sphingolipid profile was altered after myocardial infarction injury. In particular, the ratio of ceramide-1-phosphate/ceramide was increased in the myocardial infarction-injured heart tissue with a higher ceramide kinase expression (Hua et al.).

The function and effects of metabolic enzymes of fatty acids are an important topic in the field of bioactive lipids. Cytochrome P450 omega-hydroxylase 4a14 (Cyp4a14), a homolog of human CYP4A hydroxylase, catalyzes arachidonic acid to produce 20-HETE and mediates the omega-hydroxylation of medium-chain fatty acids in mice. Li et al. found that the reduction of Cyp4a14 expression mediated cholestatic-related liver fibrosis. However, as previously reported, Cyp4a14 was increased in NASH livers and promoted NASH-related fibrosis. This process implied the complex role of cyp4a14 in various liver diseases. Whether 20-HETE is involved in the effects of Cyp4a14 on cholestatic-related liver fibrosis requires further exploration. The effects of transcription factor EB (TFEB) on lipid homeostasis, including lipid degradation and efflux, as well as the regulatory effects on lipid transporters, were summarized by Li et al. In particular, the authors emphasized the role of TFEB in atherosclerosis by regulating lipid metabolism (Li et al.).

Overall, the current Research Topic includes a range of studies and reviews on the effects of bioactive lipids on cardiovascular diseases, metabolic disorders, respiratory diseases, and endocrine disorders (Figure 1). Targeting these bioactive lipids or their metabolic enzymes may provide potential therapeutic strategies for these diseases.

## Author Contributions

All authors listed have made a substantial, direct and intellectual contribution to the work, and approved it for publication.

## Conflict of Interest

JY is a part-time employee of EicOsis LLC (Davis, California, USA). The remaining authors declare that the research was conducted in the absence of any commercial or financial relationships that could be construed as a potential conflict of interest.

## Publisher's Note

All claims expressed in this article are solely those of the authors and do not necessarily represent those of their affiliated organizations, or those of the publisher, the editors and the reviewers. Any product that may be evaluated in this article, or claim that may be made by its manufacturer, is not guaranteed or endorsed by the publisher.

